# White Matter Abnormalities Correlating with Memory and Depression in Heroin Users under Methadone Maintenance Treatment

**DOI:** 10.1371/journal.pone.0033809

**Published:** 2012-04-09

**Authors:** Wei-Che Lin, Kun-Hsien Chou, Chien-Chih Chen, Chu-Chung Huang, Hsiu-Ling Chen, Cheng-Hsien Lu, Shau-Hsuan Li, Ya-Ling Wang, Yu-Fan Cheng, Ching-Po Lin

**Affiliations:** 1 Department of Diagnostic Radiology, Chang Gung University College of Medicine, Kaohsiung Chang Gung Memorial Hospital, Kaohsiung, Taiwan; 2 Department of Biomedical Imaging and Radiological Sciences, National Yang-Ming University, Taipei, Taiwan; 3 Institute of Neuroscience, National Yang-Ming University, Taipei, Taiwan; 4 Department of Psychiatry, Chang Gung University College of Medicine, Kaohsiung Chang Gung Memorial Hospital, Kaohsiung, Taiwan; 5 Department of Neurology, Chang Gung University College of Medicine, Kaohsiung Chang Gung Memorial Hospital, Kaohsiung, Taiwan; 6 Department of Internal Medicine, Chang Gung University College of Medicine, Kaohsiung Chang Gung Memorial Hospital, Kaohsiung, Taiwan; Centre for Addiction and Mental Health, Canada

## Abstract

Methadone maintenance treatment (MMT) has elevated rates of co-morbid memory deficit and depression that are associated with higher relapse rates for substance abuse. White matter (WM) disruption in MMT patients have been reported but their impact on these co-morbidities is unknown. This study aimed to investigate changes in WM integrity of MMT subjects using diffusion tensor image (DTI), and their relationship with history of heroin and methadone use in treated opiate-dependent individuals. The association between WM integrity changes from direct group comparisons and the severity of memory deficit and depression was also investigated. Differences in WM integrity between 35 MMT patients and 23 healthy controls were evaluated using DTI with tract-based spatial statistical analysis. Differences in DTI indices correlated with diminished memory function, Beck Depression Inventory, duration of heroin use and MMT, and dose of heroin and methadone administration. Changes in WM integrity were found in several WM regions, including the temporal and frontal lobes, pons, cerebellum, and cingulum bundles. The duration of MMT was associated with declining DTI indices in the superior longitudinal fasciculus and para-hippocampus. MMT patients had more memory and emotional deficits than healthy subjects. Worse scores in both depression and memory functions were associated with altered WM integrity in the superior longitudinal fasciculus, para-hippocampus, and middle cerebellar peduncle in MMT. Patients on MMT also had significant WM differences in the reward circuit and in depression- and memory-associated regions. Correlations among decreased DTI indices, disease severity, and accumulation effects of methadone suggest that WM alterations may be involved in the psychopathology and pathophysiology of co-morbidities in MMT.

## Introduction

Methadone hydrochloride is proven advantageous as treatment for heroin dependence [1]. But despite its extensive therapeutic applications, as maintenance treatment it has prominent adverse effects on several cognitive functions, including different levels-of-processing framework of psychological memory dysfunction and depression symptoms, which in turn contribute to poorer prognosis and quality of life [2]–[4]. Increasing the understanding of the neural basis and the associated cognitive functioning that occurs in chronic heroin use, especially in methadone maintenance therapy (MMT), may help design better treatment strategies.

Patients with MMT usually have limited short-term memory, working memory [5], visuo-spatial attention [1], visual perception and visual memory [3], long-term memory like semantic verbal fluency [3], and general cognitive speed [6]. Maladaptive memories associated with drug abuse may result in relapse to drug-seeking and drug-taking behavior. Heroin-dependent individuals also have depression that far exceeds estimates for the general population, have elevated suicide risk [7], and are associated with higher rates of drug re-use [8]. Co-morbidities in heroin-dependent individuals influence long-term treatment outcomes. Unfortunately, a clear mechanism between drugs abuse, altered memories, emotion, and the corresponding neuro-anatomy remains unknown.

The neurotoxic effects of chronic exposure to opioids can lead to cell death [9]. Moreover, neural apoptotic damage caused by chronic methadone administration in mouse brain has been detected [10], which raises the possibility that cognitive deficits in MMT patients may be due to persistent injury or incomplete functional/anatomic recovery from heroin use. Morphologic alterations of the gray matter in heroin-dependent subjects have also been reported [11]–[13] but those on white matter (WM) alterations in the brain with MMT are limited [14]. The impact of WM abnormalities on clinical performance like memory deficit and depression remains unclear.

Diffusion tensor imaging (DTI) is a non-invasive technique that can explore and provide evidence that features of WM microstructure can be closely coupled with differences in cognitive functions [15], [16]. Using DTI, fiber tract integrity in the superior longitudinal fasciculus (SLF), inferior fronto-occipital fasciculus, uncinate fasciculus, para-hippocampus, and cingulum [17], [18] has been related to both memory functions and depression. Therefore, MMT patients may have specific anatomic localization of damage that involves tracts associated with depression and poor memory function. Recently, the separation of diffusivities into axial (AD) and radial (RD) components has been validated as more specific measures in the longitudinal and transverse diffusion directions [19]. Aside from common DTI indices FA and ADC that characterize fiber integrity and edema, respectively, alterations in AD suggest axonal changes in fibers whereas changes in RD indicate disruption of myelin integrity [20]. However, no neuro-imaging study has investigated WM changes by multiple diffusion indices in MMT patients and there is paucity of knowledge regarding the characteristics of WM integrity in MMT.

The present study targeted WM integrity and aimed to explore the psychopathology and pathophysiology of co-morbidities in MMT. First, the effects of substance exposure on the WM were investigated between MMT subjects and health controls using DTI-Tract-Based Spatial Statistics (TBSS). Second, WM differences from the direct group comparisons associated with severity of co-morbidities in MMT subjects were determined. Lastly, the relationships between WM integrity alteration and the effects of drugs, including duration and intensity of heroin abuse and methadone treatment, were also determined. It is hypothesized that depression and decline in memory function in MMT are associated with specific patterns of WM damage that is dependent on heroin/methadone accumulation.

## Materials and Methods

### Participants

From March 2009 to February 2010, 35 patients with heroin dependence (32 males, 3 females; median age, 37 years; range, 23–59 years) under methadone maintain therapy were prospectively enrolled at the Psychiatry Department of Chang Gung Memorial Hospital. For comparison, 23 sex- and age-matched healthy subjects (22 males, 1 female; median age, 34 years; range, 21–55 years) without a medical history of neurologic disease or psychiatric illness, alcohol or substance abuse, or head injury, and with similar levels of education were recruited from within the hospital. All of the subjects received both neuro-imaging and neuro-psychiatric evaluation. The hospital's Institutional Review Committee on Human Research approved the study and all of the participants or their next of kin or guardians were provided with comprehensive information about the study and provided written informed consent.

#### Inclusion criteria of MMT

The MMT participants met the Diagnostic and Statistical Manual of Mental Disorders (DSM-IV) criteria for opiate dependence within two years prior to the study, were on a stable dose of methadone within half a year prior to the study, and were drug-free (opioids) for the last year. They were also required to have negative urine toxicology screening tests (excluding methadone) as reported in their treatment programs. Questionnaires about years of heroin abuse, approximate dollars spent daily on heroin, years of methadone use, and typical methadone dose were collected.

#### Exclusion criteria

The exclusion criteria were current or lifetime history of any Axis I diagnosis (other than opiate dependence and nicotine use), current alcohol intake more than 15 drinks (1.5 oz liquor, 12 oz beer, or 5 oz wine equivalents) per week, history of head trauma or neurologic disease, and HIV sero-positivity. Heroin-induced depressive syndrome was the only depressive disorder history allowed. Patients who exhibited Axis I depressive illness before heroin use were also excluded, as well as potential participants with a history of recent benzodiazepine use or current diagnosis of sedative/hypnotic dependence.

The exclusion criteria for control subjects included current or remote history of significant drug abuse. However, moderate use of caffeine (<600 mg of caffeine per day based on self-reports) was acceptable. Control subjects with a history of recent benzodiazepine use and alcohol intake (more than 15 drinks per week) were also excluded.

### Neuropsychological (NP) tests

The MMT group completed the NP testing in the afternoon before daily methadone dosing. The healthy volunteers also completed theirs in the afternoon. Participants were asked not to consume alcohol or benzodiazepines for 24 hours prior to the NP tests. All participants completed their tests either before or within two days after the MRI studies.

For memory functions, verbal and non-verbal episodic memory were assessed using the *Word Sequence Learning Test* [21], and an irregular geometric memory task derived from the *Benton Visual Retention Test* [22]. Verbal semantic memory was assessed by the *Remote and Recent Life Event Test* [23] and the *Semantic Association of Verbal Fluency* [24]. Short-term memory was evaluated using the *digit forward span*, *backward span, and forward-backward span*, while executive functions were assessed by the *arithmetic, digit symbol* and *similarity* sub-tests of the *WAIS-R* [25], and by *proverbs* [26].

Language screening included *Object Naming* and *the Token Test of the NCCEA* [27] whereas visual constructional praxis was assessed by performing the *Three-Dimensional Block Construction-Model* [28] and the *block design*, *object assembly* sub-tests of the *WAIS-R*. All tests were administered to both patients and controls for statistical comparison.

The Beck Depression Inventory II (BDI) and Beck Anxiety Inventory (BAI) were 21-item self-report questionnaires used to evaluate the severity of depression and anxiety symptoms, respectively [29].

### Image acquisition

The MR data were acquired on a 3.0T whole body GE Signa MRI system (General Electric Healthcare, Milwaukee, WI, USA). A standardized protocol with an eight-channel head coil was used in all participants aside from whole brain conventional structural imaging sequence to identify any brain abnormality [3D axial FSPGR T1 weighted images and axial FSE T2 weighted images]. Diffusion-weighted images were also acquired for whole brain voxel-wise analysis on the WM microstructure. To minimize motion artifacts generated during the scan, the subject's head was immobilized with foam pillows inside the coil. Diffusion tensor imaging were acquired using a single-shot echo planar imaging sequence [repetition time (TR) = 15800 ms, echo time (TE) = 77 ms, number of excitation (NEX) = 3, matrix size = 128×128, field of view (FOV) = 25.6 cm, voxel size = 2×2×2.5 mm^3^, 55 axial oblique slices without gaps]. The diffusion images gradient encoding schemes included 13 non-collinear directions with b-value 1000 s/mm^2^ and a non-diffusion weighted image volume (null image, b-value 0 s/mm^2^). The total scanning time for each subject was approximately 20 min.

#### Data preprocessing

All DTI datasets were pre-processed with the FSL v4.1.7 (Functional Magnetic Resonance Imaging of the Brain Software Library; http://www.fmrib.ox.au.uk/fsl) and in-house software. First, the raw DTI dataset were corrected for eddy current distortion and head motion by registering the diffusion-weighted images with the null image through the affine transformations using FMRIB's Diffusion Toolbox v2.0 (FDT, part of FSL) [30]. Subsequently, DTI dataset were skull striped using Brain Extraction Tool v2.1 (BET, part of FSL) [31] to remove background noise and non-tissue components. After DTI pre-processing, parameter maps, including AD (axial diffusivity, λ1), RD (radial diffusivity, [(λ2+λ3)/2]), MD (mean diffusivity, [(λ1+λ2+λ3)/3] and FA (fractional anisotropy), were calculated by fitting Baser's DTI tensor model [32] using the in-house software.

#### White matter microstructure analysis (DTI-TBSS)

Whole-brain voxel-wise statistical analysis for the DTI parameter maps was conducted using standard Tract-Based Spatial Statistics approach (TBSS) [33]. Briefly, the FA maps of all participants were non-linear aligned to the standard FMRIB58 FA template in the Montreal Neurological Institute (MNI) 152 standard space using FMRIB's Non-linear Image Registration Tool vl .0 (FNIRT, part of FSL) [30] and re-sampled into 1 mm cubic resolution. A cross-subject mean FA image was created by averaging all normalized FA maps and then skeletonized to construct a mean WM skeleton to represent the centers of all tracts common to the entire study group. The threshold of mean WM skeleton was set as FA value of 0.2.

Each participant's normalized FA data was then projected onto the group mean WM skeleton by filling the skeleton with FA values from the nearest relevant tract center. Using the same non-linear transformation matrix, WM skeleton and skeleton projection information derived from FA maps, AD, RD and MD maps were also projected onto the WM skeleton before whole-brain voxel-wise statistical analysis across subjects.

### Statistical analysis

#### Analysis of demographic data between groups

Clinical data, including gender, history of smoking, and alcohol consumption between the two groups were analyzed using Chi-square or Fisher's exact tests, where appropriate. Age and education were compared using Student's t test. Statistical differences in NP tests between the two groups were estimated by one-way analysis of covariance (ANCOVA) with participant's age, gender, and education level as covariates. The threshold for statistical significance was *p*<0.05.

#### Analysis of group comparison on FA value

To localize WM microstructure differences in the skeletonized FA maps between the two groups, voxel-wise permutation-based non-parametric analysis of covariance (ANCOVA) with a standard general linear model design matrix was used [34]. Age, gender, years of formal education, and histories of smoking and alcohol consumption were demeaned and served as nuisance covariates to ensure that any observed difference of FA was independent of these factors. Statistical inference on each possible contrast was performed using 5000 permutations in random using Randomize v2.1 (part of FSL). The resulting statistical maps were calculated and corrected for multiple comparisons with cluster-forming threshold t>3 and cluster-wise significance level corrected *p*<0.05 [33]. The most probable fiber tracts and anatomic localization of each significant cluster were determined using the means of FSL atlas tool (http://www.fmrib.ox.ac.uk/fsl/fslview/atlas.html). The mean DTI-related indices (including AD, RD, MD, and FA) within each significant cluster were calculated for each participant and further correlated to clinical evaluations.

#### Correlation between regional DTI-related indices and clinical evaluations

The Shapiro-Wilk test used to examine the data distribution of regional DTI-related indices revealed that regional DTI-related indices satisfied the parametric assumption. Subsequently, partial Pearson correlation analysis with age, sex, and years of formal education, and histories of smoking and alcohol consumption as nuisance covariates were performed to correlate the clinical evaluations (e.g., severity of depression, memory decline, duration of heroin use and MMT, and dose of heroin and methadone administration) with the regional DTI-related indices within patient groups. The threshold for statistical significance was set at *p*<0.05 with Bonferroni's correction for multiple comparisons. All statistical analyses were performed using the SPSS software, version 10.0 (SPSS Inc, Chicago, IL).

## Results

### Clinical characteristics and cognitive profiles between groups

The mean duration of heroin use was 12.4 years (range, 1–24 years) and the age of first heroin use ranged from 12 to 58 years, with a mean of 24.9 years old. Daily heroin consumption for each subject was estimated at 0.1–4.5 g before they entered the MMT program. Participants received MMT for an average of 20.3 months (range, 6–43 months) before being scanned. The last dose of methadone prior to scanning averaged 27.7 mg (range, 5–110 mg). Except for lower educational level (*p*<0.001), there were no significant differences in age, gender, or cigarette and alcohol consumption between the two groups (Table 1).

**Table 1:** Clinical characteristics and neuropsychological assessments between the MMP and controls groups.

Group	MMP (n = 35)	Control (n = 23)	F or X^2^	*p* value
Age (years)	37.00±7.96	34.32±7.45	0.235	0.158
Gender	3F/32M	1F/22M	0.386	1.000
Education (years)	10.71±1.88	15.22±1.31	6.305	**0.000** *
Alcohol (yes)^b^	13	6	0.770	0.410
Nicotine(yes)^c^	24	11	2.496	0.170
**Memory**				
Verbal episodic memory				
*WSLT, correct*	47.23±9.16	52.78±7.50	0.183	0.671
*WSLT, position*	36.20±14.77	45.60±13.76	0.535	0.468
*WSLT, learning*	6.97±7.10	11.91±6.52	1.686	0.200
Nonverbal episodic memory				
*BVRT, correct*	12.60±2.32	14.43±0.79	0.530	0.470
*BVRT, delay*	2.74±0.10	2.91±0.13	0.703	0.405
Verbal semantic memory				
*Remote Life Events Test*	14.78±0.27	14.16±0.37	1.282	0.263
*Recent Life Events Test*	14.23±1.33	14.87±0.63	0.497	0.484
*Semantic Association of Verbal Fluency* a	11.18±0.54	13.91±3.11	4.763	**0.034** *
Short-term memory				
*Digital Span Forward (WASI-R)*	8.50±0.21	7.68±0.28	3.751	0.058
*Digital Span Backward (WASI-R)*	4.46±1.62	6.04±1.36	5.459	**0.023** *
*Digital Span Forward-Backward (WASI-R)*	3.46±2.38	2.43±1.27	5.085	**0.028** *
**Visual constructional praxis**				
*3D block construction model, correct*	28.91±0.37	29.00±0.00	0.044	0.835
*3D block construction model, correct time*	117.23±50.28	90.52±25.78	0.046	0.831
*Block design*	8.92±467	11.03±0.64	4.947	**0.030** *
*Object assembly*	6.06±2.00	9.13±2.90	2.952	0.092
**Executive Function**				
*Arithmetic (WASI-R)*	8.31±2.41	11.00±3.10	0.072	0.789
*Digit symbol coding (WASI-R)*	8.49±2.14	11.70±2.55	4.947	**0.030** *
*Similarity (WASI-R)*	6.37±1.61	8.87±1.79	0.810	0.372
*Proverbs*	7.04±0.44	9.00±1.24	5.323	**0.025** *
**Language**				
*Object Naming*	15.83±0.38	15.91±0.29	0.543	0.465
*Token Test*	11.77±1.50	11.96±0.21	0.712	0.402
**Beck Depression Inventory**	17.11±14.43	1.78±2.76	5.896	**0.019** *
**Beck Anxiety Inventory**	7.26±8.31	1.26±2.11	11.87	**0.001** *

Abbreviations: MMP, methadone maintenance patient; WASI, Wechsler Abbreviated Scale of Intelligence; WSLT, word sequence learning test correct; BVRT, Benton Visual Retention Test;

a Used to measure associative verbal fluency for animals, fruits and vegetables.

b Subjects occasionally drank alcohol during their social activities.

c Subjects smoked more than 10 cigarettes a day.

* The threshold for statistical significance was set at P<0.05.

Semantic verbal memory [Semantic Association of Verbal Fluency, (F(1, 53) = 4.763; *p* = 0.034)], short-term memory [Digit Span Backward (F(1, 53) = 5.459; *p* = 0.023), Forward-Backward (F(1, 53) = 5.085; *p* = 0.028], visual constructional [Block Design, (F(1, 53) = 4.947; *p* = 0.030)], and executive function [Digit symbol coding, (F(1, 53) = 4.947; *p* = 0.030; Proverbs, (F(1, 53) = 5.323; *p* = 0.025)] were worse in MMT patients than in normal controls. The BDI scores (F(1, 53) = 5.896; *p* = 0.019) and BAI scores (F(1, 53) = 11.87; *p* = 0.001) were higher in the MMT patients than in normal controls. There were no significant differences in episodic memory and language between groups.

### Regional WM integrity aberrances between groups

**Figure 1:**
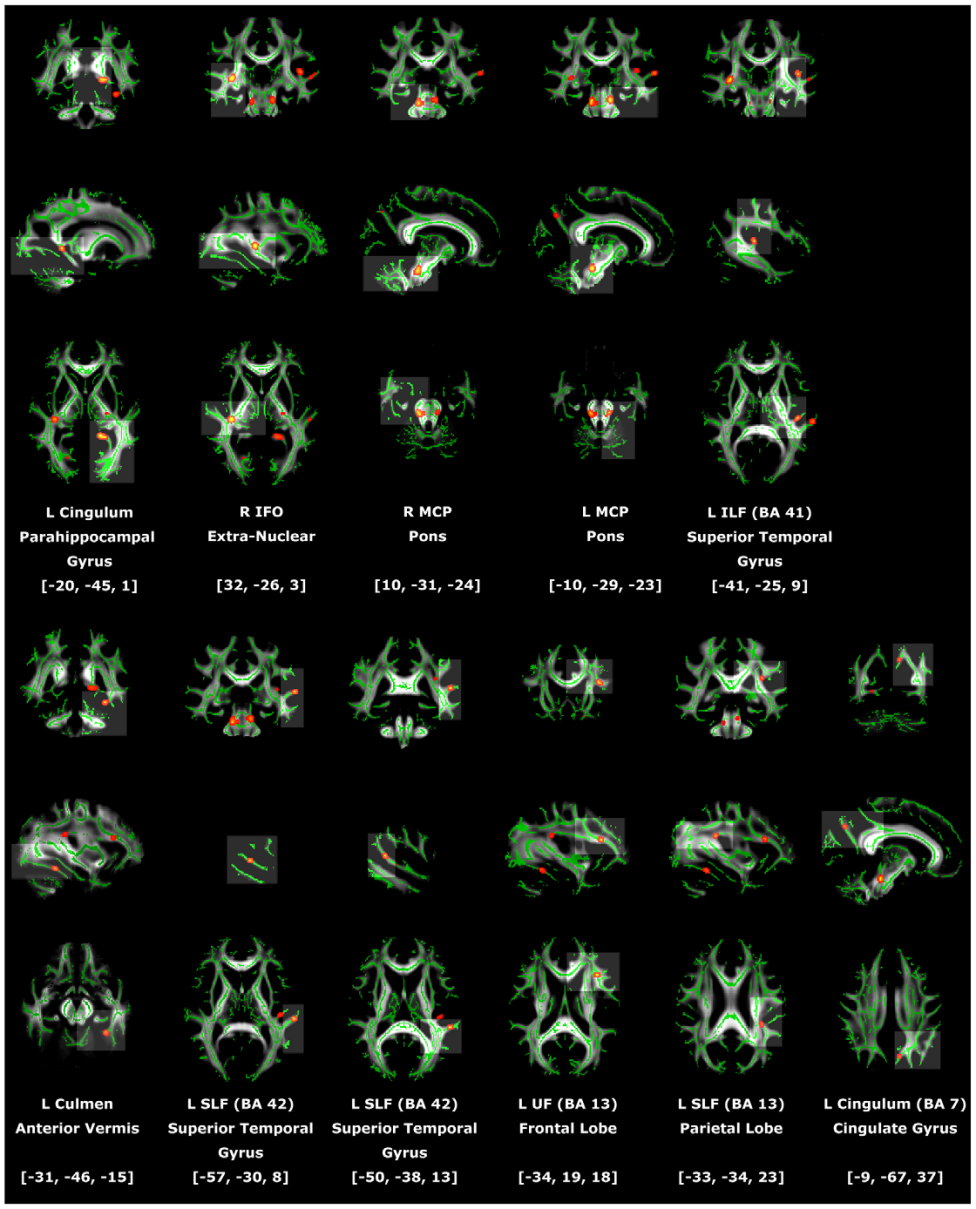
Regions with significantly lower fractional anisotropy (FA) in MMT vs. NC. All reported brain images were acquired using the “tbss_fill” script from the FSL package. The mean group FA skeleton (green) was overlaid on the mean_whole_group_FA images in axial, sagittal, and coronal views. The threshold of the mean FA skeleton was set at 0.2. Regions with significantly lower FA in MMT vs. NC were highlighted on the mean FA skeleton in colored voxels (scale range, from red to yellow). The resulting statistical maps were calculated and corrected for multiple comparisons with cluster-forming threshold t>3 and cluster-wise significance level corrected *p*<0.05. BA, Brodmann area; IFO, inferior fronto-occipital fasciculus; ILF, inferior longitudinal fasciculus; L, left; MCP, middle cerebellar peduncle; R, right; SLF, superior longitudinal fasciculus; UF, uncinate fasciculus.

Exploratory group-wise comparison between the MMT and normal groups showed that the MMT group had significantly lower FA in several WM regions. Together with decreased FA, WMs with increased RD and small or no decrease in AD were found in the left cingulum (para-hippocampus and cingulated gyrus), bilateral cerebellar peduncles (MCP), left inferior longitudinal fasciculus (ILF), left superior longitudinal fasciculus (SLF), and left uncinate fasciculus. Together with decreased FA, WMs with increased RD and decreased AD were found in the right inferior fronto-occipital fasciculus, anterior vermis of cerebellum, and left uncinate fasciculus (Table 2; Fig. 1). No regions showed decreased FA, decreased AD, or increased RD in the control group.

**Table 2:** Regions showing lower fractional anisotropy (FA) values in methadone maintenance treatment (MMT) and normal control (NC).

MNI atlas coordinates			Voxel size	White matter tract	Corresponding cortical area	FA mean (SD)		t_max_	Diffusivity values (MMT-NC)		
X	Y	Z				NC	MMT		MD	AD	RD
Decreased FA in MMT Than NC											
−20	−45	1	24	Left Cingulum	Para-hippocampal Gyrus, BA30	**0.57 (0.05)**	**0.50 (0.06)**	**3.96**	33.85	−87.43	**94.41** *
32	−26	3	20	Right Inferior Fronto-occipital Fasiculus	Extra-Nuclear	**0.65 (0.05)**	**0.59 (0.05)**	**3.86**	7.53	**−105.89** *	**64.44** *
10	−31	−24	19	Right Middle Cerebellar Peduncle	Brainstem, Pons	**0.65 (0.06)**	**0.61 (0.06)**	**4.08**	41.36	−70.16	**97.03** *
−10	−29	−23	18	Left Middle Cerebellar Peduncle	Brainstem, Pons	**0.62 (0.08)**	**0.56 (0.07)**	**4.09**	**71.36** *	−46.33	**130.29** *
−41	−25	9	17	Left Inferior Longitudinal Fasciculus	Superior Temporal Gyrus, BA41	**0.35 (0.04)**	**0.32 (0.04)**	**4.29**	24.65	−36.78	**55.15** *
−31	−46	−15	17	Left Culmen	Anterior Vermis of Cerebellum	**0.30 (0.06)**	**0.27 (0.06)**	**4.19**	−12.47	**−125.29** *	**43.93** *
−57	−30	8	16	Left Superior Longitudinal Fasciculus	Superior Temporal Gyrus, BA42	**0.34 (0.06)**	**0.30 (0.05)**	**4.40**	54.07	−13.99	**87.97** *
−50	−38	13	14	Left Superior Longitudinal Fasciculus	Superior Temporal Gyrus, BA41	**0.43 (0.05)**	**0.38 (0.06)**	**4.83**	35.02	−49.20	**77.26** *
−34	19	18	13	Left Uncinate Fasciculus	Frontal Lobe, BA13	**0.58 (0.06)**	**0.53 (0.06)**	**4.03**	25.14	−**120.08** *	**97.71** *
−33	−34	23	11	Left Superior Longitudinal Fasciculus	Parietal Lobe, BA13	**0.44 (0.06)**	**0.37 (0.04)**	**3.90**	19.69	−45.99	**52.62** *
−9	−67	37	10	Left Cingulum	Cingulate Gyrus, BA7	**0.45 (0.08)**	**0.42 (0.09)**	**4.03**	44.28	−72.40	**102.65** *

Boldfaced t_max_ values represent clusters that passed the cluster based statistical criteria with *t>3*, corrected *p*<0.05. The diffusivity values describe differences (MMT-NC) in mean diffusivity (MD), axial diffusivity (AD), and radial diffusivities (DR) (mm^2^/s) multiplied by 10^−6^.

* Significant differences (*p*≤0.05, Bonferroni-adjusted) with age, sex, education, and histories of smoking and alcohol consumption as covariates.

Abbreviations: MNI, Montreal Neurological Institute; BA, Brodmann area.

### Relationships between DTI indices and depression symptom ratings, different memory tests, duration and dose of heroin use and MMT

#### Relation between FA and Neuropsychological (NP) tests

There were significant negative correlations between BDI scores and FA value of the left MCP (r = −0.383, *p* = 0.005), left uncinate fasciculus (r = −0.285, *p* = 0.039), and left cingulum (BA 7) (r = −0.315, *p* = 0.021) (Table 3). Semantic verbal fluency correlated with the left para-hippocampus (r = 0.359, *p* = 0.008), left MCP (r = 0.337, *p* = 0.014), and left SLF (r = 0.366, *p* = 0.007), while the digit span backward correlated with the left MCP (r = 0.305, *p* = 0.025) and left SLF (r = 0.376, *p* = 0.006). The digit span forward-backward negatively correlated with the left MCP (r = −0.282, *p* = 0.041). The block design correlated with the left SLC (r = 0.289, *p* = 0.036), right anterior lobe of cerebellum (r = 0.321, *p* = 0.019), and left uncinate fasciculus (r = 0.278, *p* = 0.044).

**Table 3:** Correlation among diffusion tensor abnormalities, depression, and memory impairment after adjustments for age, sex, education, and history of smoking and alcohol use.

Clinical variable	White Matter Tract	Corresponding cortical area	Correlation (r)	P-value
***Fractional anisotropy***				
Beck Depression Inventory	L Middle Cerebellar Peduncle	L Pons	−0.383	0.005
	L Uncinate Fasciculus	L Frontal lobe, BA13	−0.285	0.039
	L Cingulum	L Cingulate Gyrus, BA 7	−0.315	0.021
Semantic Verbal Fluency	**L Cingulum**	**L Para-hippocampal Gyrus, BA30**	0.359	0.008
	**L Middle Cerebellar Peduncle**	**L Pons**	0.337	0.014
	**L Superior Longitudinal Fasciculus**	**L Superior Temporal Gyrus, BA 41**	0.366	0.007
Digit Span Backward (WASI-R)	L Middle Cerebellar Peduncle	L Pons	0.305	0.025
	L Superior Longitudinal Fasciculus	L Superior Temporal Gyrus, BA 41	0.376	0.006
Digit Span Forward Backward (WASI-R)	L Middle Cerebellar Peduncle	L Pons	−0.282	0.041
Block Design	L Superior Longitudinal Fasciculus	L Superior Temporal Gyrus, BA 41	0.289	0.036
	**L Culmen**	**L Anterior vermis of cerebellum**	0.321	0.019
	L Uncinate Fasciculus	L Frontal lobe, BA 13	0.278	0.044
***Mean diffusivity***				
Semantic Verbal Fluency	**L Middle Cerebellar Peduncle**	**L Pons**	−0.294	0.033
***Radial diffusivity***				
Beck Depression Inventory	L Middle Cerebellar Peduncle	L Pons	0.343	0.012
Semantic Verbal Fluency	**L Cingulum**	**L Para-hippocampal Gyrus, BA 30**	−0.306	0.026
	**L Middle Cerebellar Peduncle**	**L Pons**	−0.368	0.007
	**L Superior Longitudinal Fasciculus**	**L Superior Temporal Gyrus, BA 41**	−0.356	0.009
Digit Span Backward (WASI-R)	**L Superior Longitudinal Fasciculus**	**L Superior Temporal Gyrus, BA 41**	−0.314	0.022
Digit Span Forward-Backward (WASI-R)	**L Cingulum**	**L Para-hippocampal Gyrus, BA 30**	0.300	0.029
Block Design	L Middle Cerebellar Peduncle	L Pons	−0.321	0.019

The threshold for statistical significance was set at *p*<0.05 with correction for multiple comparisons. Boldfaced anatomic regions represent significant correlation between memory tests and DTI indices after further co-varying for BDI score.

#### Relation between MD and NP tests

Only *semantic verbal fluency* was significantly negatively associated with increase MD value in the left MCP (r = −0.294, *p* = 0.033).

#### Relation between AD and NP tests

No domains showed correlation with AD.

#### Relation between RD and NP tests

The BDI scores correlated with RD in the left MCP (r = 0.343, *p* = 0.012), while the semantic verbal fluency inversely correlated with the left para-hippocampus (r = −0.306, *p* = 0.026), left MCP (r = −0.368, *p* = 0.007), and left SLF (r = −0.356, *p* = 0.009). The digit span backward inversely correlated with the left SLF (r = −0.314, *p* = 0.022), whereas scores of forward-backward correlated with the left para-hippocampus (r = 0.300, *p* = 0.029). The block design negatively correlated with the left MCP (r = −0.321, *p* = 0.019).

#### Impact of depression on correlations between memory tests and DTI indices

Further analysis between DTI indices and the aforementioned memory tests showed correlation with DTI indices after co-varying for BDI score. The significant independent relationships of memory tests with DTI indices of multiple regions remained (Table 3, boldface anatomic regions).

#### Duration and dose of heroin use and MMT vs. DTI indices

**Figure 2:**
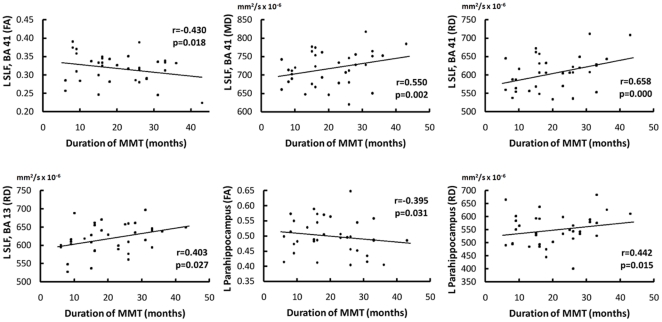
Correlations between duration of MMT and DTI indices in the left superior longitudinal fasciculus (SLF) and left para-hippocampus (adjusted for age, sex, education, and history of smoking and alcohol use).

Partial correlation analyses revealed that increased duration of MMT was associated with decreased DTI indices in the left SLF (BA 41 and 13) and left para-hippocampus (Fig. 2). Analysis between DTI indices and the duration and dose of heroin use and dose of MMT did not reveal any significant effects.

## Discussion

This study has several major findings. First, chronic heroin users under MMT have heterogeneous disruption of the WM tract, as revealed by multiple tensor metrics (FA, MD, AD, and RD). Second, there is a correlation between the duration of MMT and the extent of WM changes, which indicates that WM disruption is directly or indirectly caused by methadone. Lastly, the depression disorder and several memory and cognition tests deficits in the MMT group are consistent with previous findings [1], [2]. Differential regional WM deficits are significantly correlated to scores on these tests, revealing a direct relationship between impaired cognitive functions and WM abnormalities. Such results may shed the light in establishing a possible causal relationship among reward behavior, emotion control, and memory retrieval system in drug dependence.

By using DTI method, previous studies that WM changes occur in MMT are confirmed. Fractional anisotropy is derived from the directional diffusivities of the diffusion tensor. It decreases after either an increase in RD or a decrease in AD. In the present study, decreased FA, increased RD, and a much smaller or no change in AD, such as the left cingulum (para-hippocampus and cingulated gyrus), MCP, left ILF, left SLF, and left uncinate fasciculus (Table 2) have been suggested as myelin injury [20]. However, a decrease in FA with a reduction in AD but an increase in RD in the right inferior fronto-occipital fasciculus, anterior vermis of cerebellum, and left uncinate fasciculus, may likewise be associated with the axonal damage and demyelination or fiber reorganization found in neuro-degenerative diseases [35]. Distinctive WM disruption profiles demonstrate heterogeneous pathologic processes affecting different WM in MMT. However, their contributions to DTI changes may not be fully resolved in this cross-sectional analysis. Different WM integrity alterations may be the end results of interactions among current MMT, former heroin use, and the vulnerability of brain tissue.

Evaluation of multiple DTI indices helps to explore the histologic findings of WM and possible etiologies of disease evolution *in vivo*. Results of changes in multiple DTI indices are not consistent with those of a previous report where there are WM integrity alterations in FA values without changes in AD and RD values [14]. Vacuolar leucoencephalopathy, which attribute restricted water diffusion caused by fluid entrapment within the myelin lamellae without demyelination, may explain the previous findings [36]. However, the longer duration of heroin addiction and methadone use in the present study can result in more severe WM damage. Based on the current findings, it is possible that a progressive pattern of direct WM injury occurs in MMT, from swelling (decreased FA) to demyelination (decreased FA, increased RD) with subsequent axonal damage (decreased FA, decreased AD, and increased RD).

Pathologic evidence on WM damage also supports the current findings [10], [37]. The WM of MMT patients may exhibit vacuoles within the myelin sheaths, as well as vacuolar degeneration of axons with subsequent demyelination and/or axonal damage. Moreover, there is significant correlation between RD (index for myelin injury) and the cumulative effect of methadone. Cognitive scores encourage the important role of WM damage in the pathologic process involved in methadone use. However, decreased cortical volume [11], [12], [13] suggests that WM disruption can also be caused by neuronal damage [38]. Further validation via chronologic animal model is indicated.

Another important finding in the present study is the strong correlations among WM changes in depression, memory-associated anatomy, and severity of co-morbidities. There is considerable evidence in literature that the administration of opiates like morphine, heroin, and methadone leads to memory deficits in animals [39]. Changes in DTI indices in the left SLF and left para-hippocampus (cingulum, BA 30) are associated with different aspects of memory impairment. The SLF is a long, dorsal tract connecting fundamental regions within the language and memory networks [40]. It mainly connects the frontal, parietal, occipital, and temporal lobes. Defects of SLF in MMT patients explain their semantic memory deficit since it depends crucially on a system linking the hippocampi with the temporal and frontal lobes.

Damage to the para-hippocampus (cingulum, BA 30) and their interaction with distinct brain memory systems have also been demonstrated in the use of other substances [41]. The para-hippocampus receives input from the cingulate cortex and projects output to the entorhinal cortex, which functions as a hub in a widespread network for memory and navigation. Moroni et al. demonstrated that memory deficits may result from the apparent ability of morphine to decrease acetylcholine turnover in the hippocampus [42]. Morphine exposure also reportedly up-regulates neuronal glucocorticoid receptors [43] that have been suggested as major causative factors in hippocampal shrinkage in depression [44]. Results of the present study show that multiple regional WM integrity changes in the para-hippocampus, SLF, and especially the temporal lobe, together with other WM changes, suggest that influences to other levels of the memory process is inevitable in MMT.

In addition to memory function-associated cortex, there are also DTI changes in cortico-cortical connection fibers. WM deficits are found in the frontal lobe (uncinate fasciculus, BA 13), cerebellum (anterior vermis), and cingulated cortex, and their connection fibers in the pons of MMT patients. Most of these findings are associated with alterations in the reward circuit characteristic of drug dependence [45] and also constitute their emotion deficits [46], [47]. The uncinate fasciculus connects the hippocampus, amygdala, and orbito-frontal cortex. These structures are prominently implicated in emotion regulation and depression [48]. Deficits in the uncinate fasciculus, especially in the insular portion (BA 13), may also impair the processing of self-induced and/or internally generated emotional recall [49]. In the current study, deficits in the uncinate fasciculus and their association with poor BDI scores are consistent with inappropriate or inefficient engagement of the emotion regulatory circuitry in MMT patients.

The WM of the cerebellum and middle cerebellar peduncle are also altered in MMT patients. Several human neuro-imaging studies report cerebellar involvement in the reactivation of drug-conditioned emotional memories [50]. Drug-induced molecular and cellular changes in the cerebellum may re-organize the fronto-cerebellar networks and contribute to the addictive behavioral phenotype. DTI integrity change in the cerebellar peduncle may disconnect this circuit with the subsequently altered cerebellar neuro-plasticity in consolidating mood-related memory and cognitive procedural learning [50], resulting in depression. Cerebellar peduncles play a pivotal role in the completeness of the circuit of emotion and memory between the cerebrum and cerebellum in MMT.

Cerebellar peduncle abnormalities, together with those of the SLF and para-hippocampus, are associated with worse scores of both depression and memory functions in MMT (Table 3), which emphasize their role in modulating both memory and emotion. Further analysis shows that declined DTI indices in WM are independently associated with memory decline after co-varying the depression score. It also demonstrates that DTI integrity changes in those regions have higher contribution to memory deficits than to depression in MMT. Further investigation on their causal relationship is warranted.

Another important finding is that changes in WM integrity in the SLF and para-hippocampus are associated with the duration of MMT but not heroin use. This is consistent with a previous study [14] wherein accumulated methadone itself may have an adverse impact on WM integrity but does not eliminate WM impairment due to long-term heroin use. The correlation between methadone therapy and DTI indices further provide a possible explanation for the memory deficits. Taken together, the results suggest that DTI indices in SLF on the temporal lobe and para-hippocampus may be clinico-pathologic markers of WM change in MMT.

### Limitations

This cross-sectional study has some limitations. The most important gap is the causal-relationships among heroin, methadone, and WM change. Whether or not heroin alone or with methadone can result in WM change with subsequent cognitive impairment is not answered. The effect of methadone on memory and emotion, and its relationship with anatomic deficits can be clarified by comparing heroin dependent abstainers with and without methadone replacement treatment. Moreover, the results may be confounded by a history of use of other substances or heroin re-use in the MMT group. Lastly, it is also impossible to examine the effects of pre-existing major psychiatric illnesses and their corresponding anatomic defects that result in drug use behavior.

### Conclusions

There are significant WM differences between MMT subjects and healthy controls. Impaired neuro-circuit of the reward system in heroin users also constitutes a disconnection in depression and memory-associated regions. Correlations between WM abnormalities and the severity of depression and memory dysfunction support the involvement of WM alteration in the psychopathology and pathophysiology of these two major co-morbidities in MMT. The negative correlation between DTI indices in the SLF and para-hippocampal gyrus and the duration of methadone use suggests that DTI deficits in these WM regions may be specific biomarkers that may help improve long-term outcomes in chronic heroin users and guide methadone treatment strategies in the future.
